# Tumor Regulatory Effect of 15-Hydroxyprostaglandin Dehydrogenase (HPGD) in Triple-Negative Breast Cancer

**DOI:** 10.3390/ijms26051912

**Published:** 2025-02-23

**Authors:** Joselyn Padilla, Bok-Soon Lee, Allen Kim, Yea-In Park, Avani Bansal, Jiyoung Lee

**Affiliations:** 1 Department of Biochemistry and Molecular Medicine, School of Medicine and Health Sciences, The George Washington University, Washington, DC 20037, USA; joselynp@gwu.edu (J.P.); yeainp@gwu.edu (Y.-I.P.);; 2 Department of Otolaryngology, Ajou University School of Medicine, Suwon 16499, Republic of Korea; 3 GW Cancer Center, School of Medicine and Health Sciences, The George Washington University, Washington, DC 20037, USA

**Keywords:** triple-negative breast cancer, Prostaglandin, PDGH, 15-hydroxyprostaglandin dehydrogenase, HPGD

## Abstract

Prostaglandin regulation is known to play a pivotal role in tumorigenesis; however, the contributions of the prostaglandin-metabolizing enzyme 15-hydroxyprostaglandin dehydrogenase (HPGD) to cancer development remain poorly understood. In this study, we investigate the effects of HPGD on cell viability, proliferation, anchorage-independent growth, and migration in triple-negative breast cancer (TNBC), an aggressive subtype of breast cancer. Overexpression of HPGD in human TNBC cells resulted in both positive and negative regulation of cell proliferation and colony formation, with these effects occurring independent of prostaglandin E2 (PGE_2_). In contrast, overexpression of the mouse homolog, Hpgd, in murine TNBC cells led to a consistent but modest reduction in cell viability and colony formation, indicating that HPGD activity varies depending on species and cell line context. Notably, TNBC cells expressing a mutant form of Hpgd (Hpgd^mut^), which lacks the ability to bind PGE_2_, exhibited similar functional outcomes in cell viability and colony formation as those expressing wild-type Hpgd (Hpgd^WT^). These findings suggest that HPGD may exert its tumorigenic effects through non-enzymatic mechanisms, potentially by involving modulation of KRAS signaling in human TNBC cells. Our results highlight the diverse roles of HPGD in cancer biology, particularly in the context of TNBC, and point to non-enzymatic pathways as a significant aspect of its tumorigenic activity.

## 1. Introduction

Breast cancer is the most prevalent malignancy and a leading cause of cancer-related mortality globally [1]. Among its subtypes, triple-negative breast cancer (TNBC) is the most aggressive and difficult to treat [2]. Characterized by the absence of estrogen receptor (ER), progesterone receptor (PR), and human epidermal growth factor receptor 2 (HER2) expression, TNBC often exhibits poorer prognosis, higher recurrence rates, and resistance to conventional therapies, underscoring the urgent need for novel therapeutic strategies and more effective clinical interventions.

Dysregulated inflammation plays a crucial role in tumor progression, including breast cancer [3]. Prostaglandins—bioactive lipid mediators produced during inflammatory responses—are key modulators of various tumorigenic processes, such as cell proliferation, survival, angiogenesis, and immune evasion [4,5,6,7]. Among prostaglandins, prostaglandin E2 (PGE_2_) is particularly implicated in promoting a pro-tumorigenic microenvironment, facilitating tumor growth, invasion, and metastasis in breast cancer. For PGE_2_ homeostasis regulation, the enzyme 15-hydroxyprostaglandin dehydrogenase (HPGD, also known as 15-PGDH) catalyzes the oxidative conversion of PGE_2_ to the inactive keto forms [4]. As such, HPGD has been shown to regulate the local levels of PGE_2_, suppressing its tumor-promoting effects. In breast cancer, HPGD expression has been positively associated with estrogen receptor (ER) and tumor stages, suggesting a potential tumor-promoting role [8]. Conversely, HPGD expression is found to be low in basal and HER-2 positive subtypes and intermediate in Luminal A and B tumors, but high in normal breast [9]. Notably, reduced or silenced expression of HPGD in breast cancer leads to elevated PGE_2_ levels and enhanced tumor progression, supporting its role as a tumor suppressor [10]. However, other studies have shown that low HPGD expression correlates with poor prognosis [11], increased cancer invasion, migration [12], and resistance to certain therapies, further complicating its role as a potential tumor suppressor or promoter. This ambiguity emphasizes the need for a more comprehensive understanding of HPGD’s function in breast cancer, particularly in TNBC, where its precise role remains poorly defined.

In this study, we investigate the functional roles of HPGD in the regulation of tumor growth and phenotypic changes in TNBC, focusing on both human and murine TNBC cell lines following ectopic HPGD expression. Our findings demonstrate that HPGD’s effects on cell proliferation and tumorigenic behaviors are cell line- and species-dependent, independent of PGE_2_ levels. Overexpression of HPGD in human TNBC cells (MDA-MB231) promoted cell proliferation, while no significant effect was observed in MDA-MB436 or MDA-MB468 cells. Specifically, overexpression of Hpgd in murine TNBC cells, including 4T1.2, E0771, EMT6, and Py8119, has either modestly reduced or not affected cell proliferation. Further analyses suggest that HPGD may regulate cell growth through modulation of KRAS signaling pathways in a cell-specific manner. Together, these findings reveal that HPGD exerts diverse, non-enzymatic effects on TNBC tumorigenesis, offering new insights into its complex role in breast cancer biology.

## 2. Results

### 2.1. HPGD Regulates Proliferation of Human TNBC in a Cell Line-Dependent Manner

**Figure 1:**
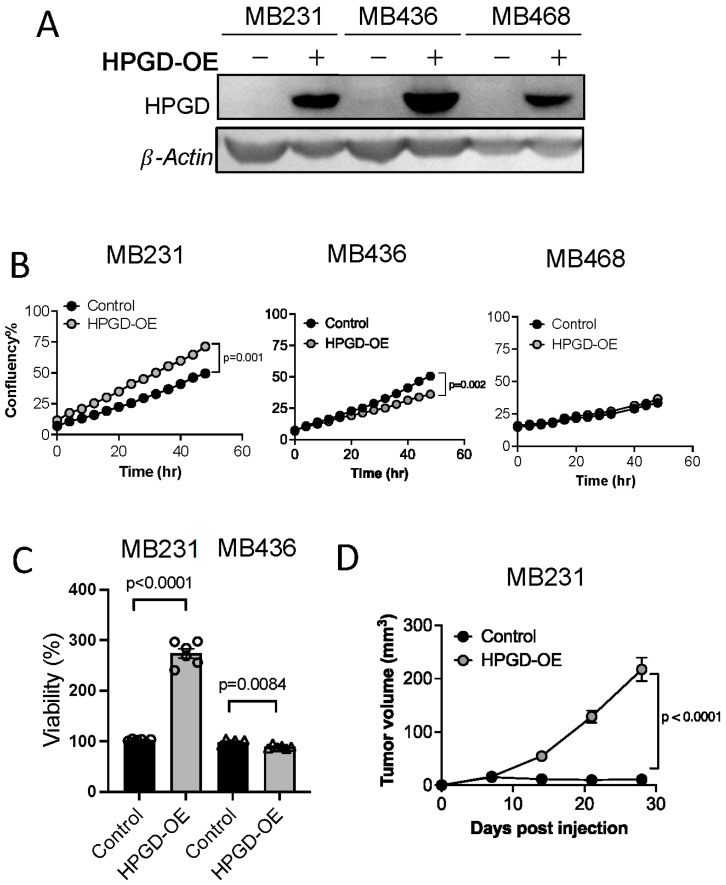
HPGD regulates the proliferation of human TNBC cells in a cell line-dependent manner. (**A**) Representative Western blot of ectopic HPGD expression using lentiviral transduction followed by 10 days of puromycin selection (2 μg/mL) in human TNBC cell lines. (**B**) Growth (% confluency) of TNBC cells overexpressing HPGD (HPGD-OE). (**C**) Viability (% live cells relative to control) of HPGD-OE TNBC cells by MTT assay (*n* = 6 biological replicates from a 96-well plate). (**D**) Tumor volume of mice xenografted using MB231-HPGD-OE and control (MB231-Control) cells as indicated (*n* = 20 mice per group). Data are mean ± SEM. *p*-values using a two-tailed, paired Student’s *t*-test.

To investigate the role of 15-hydroxyprostaglandin dehydrogenase (HPGD) in regulating cell proliferation and tumor growth, we stably transduced three human triple-negative breast cancer (TNBC) cell lines—MDA-MB231, MDA-MB436, and MDA-MB468—with lentiviral vectors carrying HPGD. Successful transduction was selected using puromycin (2 μg/mL) for 10 days. Western blot analysis confirmed the overexpression of HPGD in all three cell lines, with levels significantly higher than those in control cells (Figure 1A, Supplementary Figure S1A).

We next evaluated the impact of HPGD overexpression on cell growth by measuring percent confluency in HPGD-overexpressing cells (MDA-MB231-HPGD, MDA-MB436-HPGD, and MDA-MB468-HPGD) and their respective control counterparts. MDA-MB231-HPGD (MB231-HPGD) cells exhibited significantly higher growth rates compared to control cells, while MDA-MB436-HPGD (MB436-HPGD) demonstrated a reduction in growth rates. Interestingly, MDA-MB468-HPGD (MB468-HPGD) cells displayed growth rates comparable to control cells (Figure 1B, Supplementary Figure S1B). Cell viability, as measured by MTT assays, corroborated these findings. Specifically, MB231-HPGD cells demonstrated a markedly increased viability compared to control cells, whereas MB436-HPGD cells showed a slight but statistically significant decrease in viability relative to control cells (Figure 1C). To further investigate the role of HPGD in tumor growth in vivo, we performed xenograft experiments with MB231-HPGD or control cells. Tumor volume measurements revealed a significant increase in tumor growth in mice bearing MB231-HPGD tumors compared to control tumors (*n* = 20 per group), beginning at day 7 post-injection. By day 21, the tumors in the HPGD-overexpressing group were notably larger, indicating that HPGD overexpression enhances tumor growth in this xenograft model (Figure 1D).

### 2.2. Hpgd Decreases Proliferation of Murine TNBC Cells

**Figure 2:**
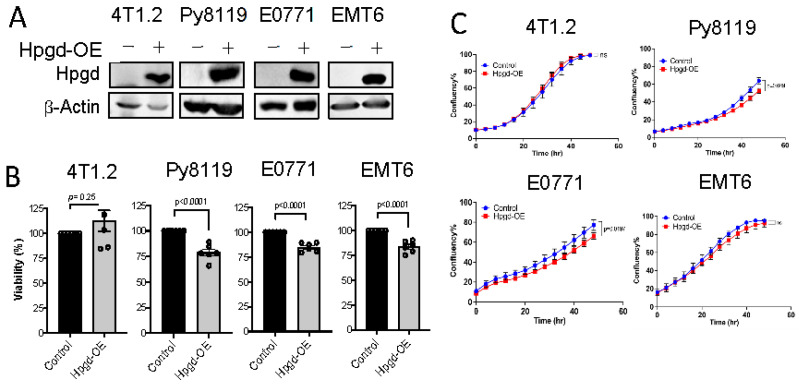

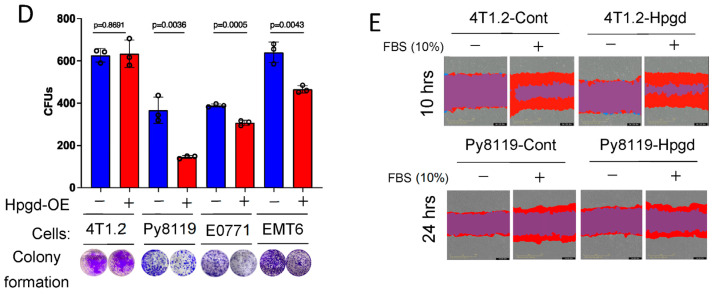
Hpgd slightly decreases the viability of murine TNBC cells. (**A**) Representative Western blot of ectopic Hpgd expression in murine TNBC cell lines. (**B**) Viability (% live cells relative to control) of Hpgd-OE cells for 48 h by MTT assay. (**C**) Growth (% confluency) of Hpgd-OE cells by Incucyte Imager. (**D**) Colony formation shown as colony-forming units (CFUs) of Hpgd-OE cells (*n* = 3 biological replicates) and representative colony images. (**E**) Representative images of migrated cells using scratch wound assays for Hpgd-expressing or control cells (marked in red) with FBS (10%) (*n* = 6 biological replicates). Data are mean ± SD. *p*-values using a two-tailed, paired Student’s *t*-test.

To further investigate the functional roles of Hpgd in tumorigenesis, we utilized murine TNBC cell lines, including 4T1.2, Py8119, E0771, and EMT6. Stable cell lines expressing Hpgd were generated through lentiviral transduction, followed by selection with hygromycin (200 μg/mL) for 10 days. Western blot analysis confirmed robust overexpression of Hpgd in all four cell lines compared to their control counterparts, indicating effective transduction and stable integration (Figure 2A). We next evaluated the effect of Hpgd on cell viability and growth using MTT assays and Incucyte Live Cell Imaging, respectively. In Hpgd-overexpressing (Hpgd-OE) Py8119, EMT6 and E0771 cells, cell viability was slightly decreased, while no significant changes in viability were observed in 4T1.2 cells (Figure 2B, Supplementary Figure S2A). Similar results were obtained in growth assays, where Hpgd-OE led to a modest reduction in cell growth in E0711 and Py8119, but no effect was observed in 4T1.2 and EMT6 cells (Figure 2C, Supplementary Figure S2B). We further investigated the impact of Hpgd on colony-forming ability, which provides insights into the single cell’s potential to proliferate and survive over a prolonged time as a self-renewal and tumor-initiating property. Consistent with the viability and growth assays, colony formation was remarkably decreased in Py8119, E0771, and EMT6 cells, while 4T1.2 cells showed no change in colony formation capacity (Figure 2D, Supplementary Figure S2C). Furthermore, to assess whether Hpgd affects the migratory properties of these cells, we performed scratch wound assays using 4T1.2-Hpgd and Py8119-Hpgd cells along with their respective controls. The non-migrative murine TNBC cell line E0771 was excluded from migration assays. Migration assays, including scratch wounds, commonly assess the ability of cancer cells’ mobility, like cancer metastasis. Real-time monitoring of wound closure revealed no significant difference in migration between Hpgd-OE and control cells, suggesting that Hpgd does not impact cell migration in murine TNBC cells (Figure 2E, Supplementary Figure S2D). Taken together, these results indicate that ectopic expression of Hpgd leads to a modest reduction in cell viability, growth, and colony formation in certain murine TNBC cell lines. However, Hpgd does not appear to affect cell migration, highlighting its limited functional role in murine TNBC tumorigenesis.

### 2.3. HPGD Regulates Spheroid Formation in a Cell Line-Dependent Manner

**Figure 3:**
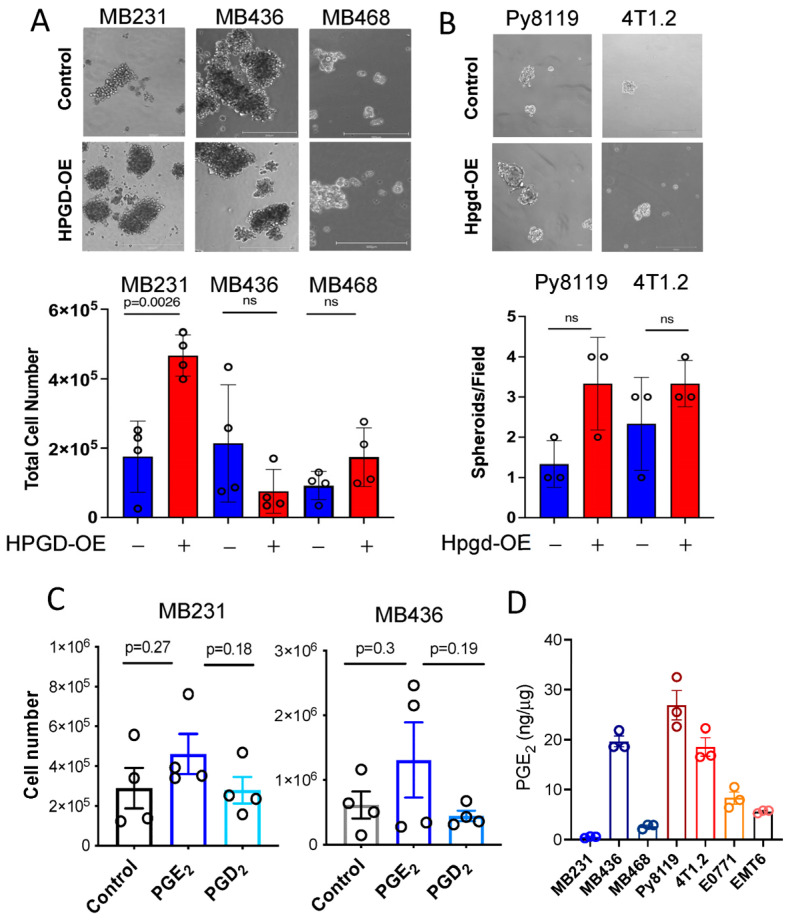
HPGD regulates spheroid formation of TNBC cells in a cell line-dependent manner. (**A**,**B**) Representative images of spheroid formation and total cell numbers from spheroids of human cell lines (**A**) and murine cell lines (**B**) expressing ectopic HPGD and cultured in 3D mammosphere media for 7–10 days. The scale bar is 300 μm. (**C**) Total cell numbers from spheroids of MB231 and MB436 cells treated with exogenous PGE_2_ or PGD_2_ in 3D mammosphere media for 10 days (*n* = 4 biological replicates). (**D**) Endogenous PGE_2_ levels of human and murine TNBC cells. PGE_2_ levels normalized to whole cell protein concentration (*n* = 3 technical replicates). Data are mean ± SD for (**A**,**B**), or mean ± SEM for (**C**,**D**). *p*-values using a two-tailed Student’s *t*-test. ns indicates not significant (*p* > 0.05).

To better represent the effects of HPGD on cell growth in a more physiologically relevant context, we employed three-dimensional (3D) spheroid formation assays, which serve as a model for breast tumor growth [13,14,15]. Using specialized 3D spheroid culture media, MB231, MB436, and MB468 cells overexpressing HPGD (HPGD-OE) or control cells were cultured for 10 days. Spheroids formation was then imaged and analyzed for size and cell numbers. In MB231 cells, HPGD overexpression led to significantly larger spheroids compared to control cells (Figure 3A, Supplementary Figure S3A). Moreover, when total cell numbers within the spheroids were quantified after spheroid spin-down and resuspension into single-cell suspensions, MB231-HPGD spheroids exhibited significantly higher cell counts than their control counterparts. In contrast, spheroid size and total cell numbers in MB436 and MB468 cells were comparable between HPGD-OE and control cells, despite ectopic expression of HPGD (Figure 3A). These findings align with results from 2D cell growth assays, where HPGD’s effects on proliferation were cell line-dependent (Figure 1B,C). We further assessed the effect of Hpgd overexpression on spheroid formation in murine cell lines, including 4T1.2 and Py8119. Interestingly, Hpgd overexpression did not result in significant differences in spheroid formation compared to control cells (Figure 3B). Both the number of spheroids bigger than 50 cells and their size (diameter and total cell count) were similar between Hpgd-OE and control cells (Supplementary Figure S3B). These results indicate that HPGD regulates spheroid formation in a cell line-dependent manner in human TNBC cells, while Hpgd has a limited or absent role in the 3D growth of murine TNBC cells.

### 2.4. HPGD Regulation of Growth and Sphere Formation Is Independent of PGE2

Given that the canonical function of HPGD involves the downregulation of PGE_2_, we hypothesized that the growth regulatory effect of HPGD in MB231 and MB436 might be dependent on PGE_2_ levels. To test this, we supplemented the culture medium of MB231 and MB436 cells with exogenous PGE_2_ (500 ng) and assessed their ability to form spheroids in 3D culture. Surprisingly, exogenous PGE_2_ supplementation had no significant effect on spheroid formation in either MB231 or MB436 cells. Similarly, PGD_2_, a prostaglandin analog control that is not regulated by HPGD, also did not affect spheroid formation in these cell lines (Figure 3C). Furthermore, we evaluated the basal levels of PGE_2_ in the cell lines used in this study. Despite the generally low expression of HPGD in breast cancer cells, endogenous PGE_2_ levels varied in a cell line-dependent manner (Figure 3D). Notably, MB231 exhibited the lowest levels of endogenous PGE_2_ among the tested lines. In addition, we observed that HPGD overexpression had differential effects on PGE_2_ levels across the human TNBC cell lines. Specifically, in MB231 cells, HPGD overexpression resulted in a significant increase in PGE_2_ levels, while in MB436 and MB468 cells, HPGD overexpression led to a slight reduction in PGE_2_ levels. This suggests that MB231 cells may regulate PGE_2_ homeostasis through negative feedback mechanisms (Supplementary Figure S3C). Collectively, these findings suggest that the growth regulatory effects of HPGD, including its impact on spheroid formation, do not directly depend on PGE_2_ levels, indicating that HPGD may exert its effects through alternative, PGE_2_-independent mechanisms.

### 2.5. HPGD’s Enzymatic Function Does Not Affect Cell Proliferation

**Figure 4:**
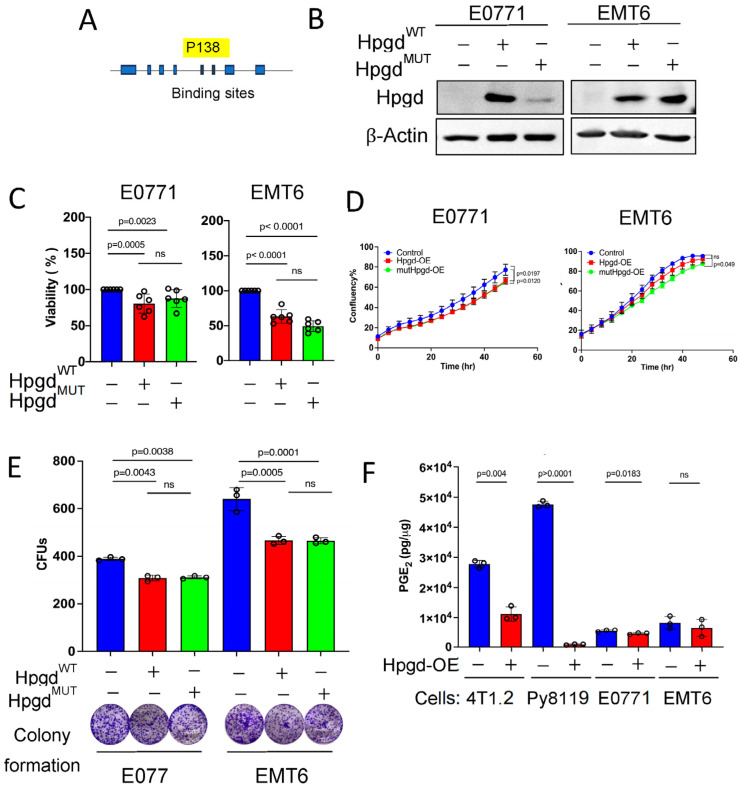
The enzymatic function of Hpgd is not required for the regulation of cell proliferation. (**A**) Schematic of mutation at the substrate binding site of Hpgd (269 aa). (**B**) Representative Western blot of Hpgd^WT^ and mutant (Hpgd^mut^) expression in stable E0771 and EMT6 cell lines. (**C**) Viability (% live cells relative to control) of cells overexpressing Hpgd^WT^, Hpgd^mut^, or control vector using Calcein AM assays (*n* = 6 biological replicates). (**D**) Growth (% confluency) of cells overexpressing Hpgd^WT^, Hpgd^mut^, or control vector for 48 h using Incucyte (*n* = 6 biological replicates). (**E**) Colony-forming units (CFUs) of cells overexpressing Hpgd^WT^, Hpgd^mut^, or control vector using colony formation assays with representative colony images (*n* = 3–9 biological replicates). (**F**) Endogenous PGE_2_ levels of murine TNBC cells-overexpressing Hpgd. PGE_2_ levels normalized to whole cell protein concentration (n=3 technical replicates). Data are mean ± SD. *p*-values using a two-tailed Student’s *t*-test. ns indicates not significant (*p* > 0.05).

To determine whether the non-canonical functions of HPGD are critical for tumorigenesis independent of PGE_2_ levels, we generated a mutation in the PGE_2_-binding domain of Hpgd (Figure 4A). Given that exogenous PGE_2_ supplementation did not affect spheroid formation of human TNBC cells, we focused on mutating murine Hpgd for expression in murine TNBC cells. A proline (P) to alanine (A) substitution at position 138 (P138A) in the catalytic domain of Hpgd was introduced, which abolishes PGE_2_ binding and enzymatic activity [16]. We then constructed stable cell lines expressing either the mutated Hpgd (Hpgd^P138A/^Hpgd^mut^) or wild-type Hpgd (Hpgd^WT^) using lentiviral transduction and hygromycin selection (200 µg/mL) for 10 days. Successful overexpression of Hpgd and Hpgd^mut^ was confirmed in E0771-Hpgd^mut^ and EMT6-Hpgd^mut^ cells, alongside Hpgd^WT^-expressing cells (Figure 4B). Using these stable cell lines, we assessed the impact of Hpgd^mut^ on cell proliferation through cell viability and growth assays. The results showed that cells expressing Hpgd^mut^ exhibited a similar suppression of cell viability as those overexpressing Hpgd^WT^ in both E0771 and EMT6 cells (Figure 4C, Supplementary Figure S4A). Additionally, cell growth as measured by confluency showed no significant difference between Hpgd^mut^ and Hpgd^WT^-expressing cells, with both groups demonstrating a modest decrease in cell growth (Figure 4D, Supplementary Figure S4B). Colony formation assays also revealed that the number and size of colonies were comparable between Hpgd^mut^ and Hpgd^WT^-expressing cells (Figure 4E, Supplementary Figure S4C). These results indicate that both Hpgd^mut^ and Hpgd^WT^-expressing cells exhibit decreased cell growth and viability compared to controls. Importantly, the absence of enzymatic activity in the Hpgd^mut^-expressing cells suggests that HPGD’s non-canonical function, rather than its enzymatic function or substrate binding, plays a key role in regulating cell proliferation in these cell lines.

### 2.6. HPGD Modulates KRAS Signaling Pathway

**Figure 5:**
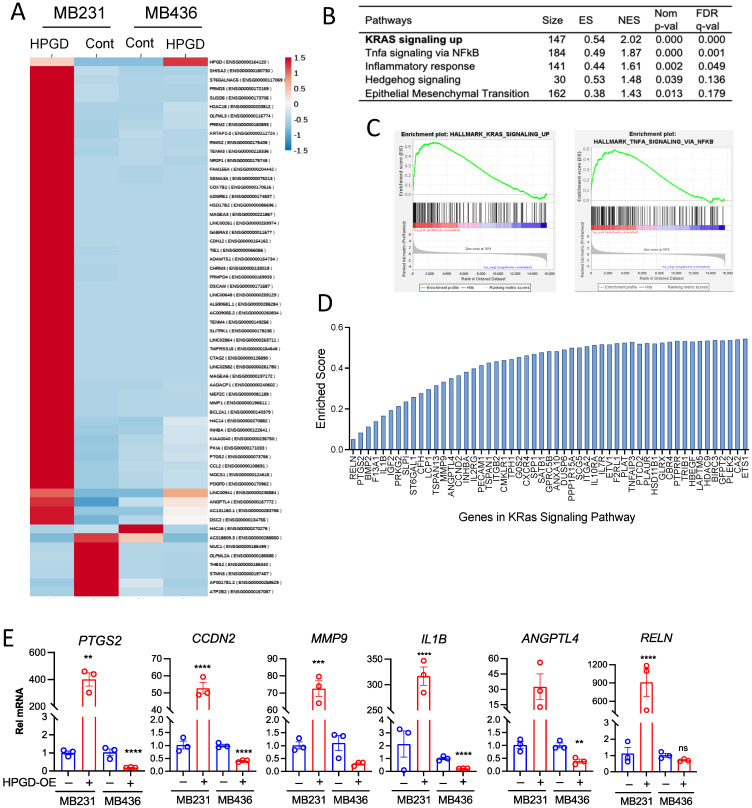
HPGD positively regulates gene expression associated with KRAS signaling in MB231 cells, whereas it exerts a negative regulatory effect on gene expression in MB436 cells. (**A**) Heat map of genes differentially expressed in MB231-HPGD and MB436-HPGD cells. (**B**) GSEA indicates functional pathways regulated by HPGD in MB231 cells. Adjusted *q*-values (FDR) are shown. (**C**) Enrichment scores (ES), normalized enrichment score (NES), normalized *p*-values, FDR q-values of KRAS signaling and TNF alpha signaling by HPGD in MB231 cells. (**D**) Genes listed with ES in the KRAS signaling pathway. (**E**) Relative mRNA expression levels of MB231-HPGD and MB436-HPGD cells by qRT-PCR. *p*-values by unpaired *t*-test. ** *p* < 0.01, *** *p* < 0.001, **** *p* < 0.0001.

Given that HPGD regulates cell growth and viability in human TNBC cells independent of PGE_2_ levels, we sought to further investigate the downstream signaling pathways affected by HPGD. To this end, we performed bulk RNA sequencing using stable HPGD-expressing MB231 and MB436 cells and control cells. Transcriptomic analysis of RNA sequencing data revealed that the changes in gene expression were more dependent on the cell line itself than on HPGD expression (Figure 5A). Heatmap analysis demonstrated that HPGD primarily regulated gene expression in MB231 cells, while its effect on gene expression in MB436 was minimal. Since gene expression changes in MB436 cells did not show statistically robust alterations upon HPGD overexpression, we excluded MB436 for further analysis and focused on the data derived from MB231-HPGD cells.

To further explore the functional pathways by HPGD, we performed Gene Set Enrichment Analysis (GSEA) on MB231-HPGD and control cells. Among the top pathways identified were Kristen Rat Sarcoma Virus (KRAS) signaling, Tumor Necrosis Factor (TNF) alpha signaling via Nuclear Factor Kappa B subunit (NFkB), and inflammatory responses, all of which showed statistical significance (FDR *q* < 0.05) and enriched scores (ES) (Figure 5B–D). In particular, KRAS signaling was found to be a major pathway affected by HPGD. To validate these findings, we focused on genes associated with KRAS signaling as identified by GSEA and measured their expression using quantitative RT-PCR (Figure 5E). We observed that KRAS signaling-associated genes, including *RELN*, *PTGS2*, *CCDN2*, *MMP9*, *IL1B*, and *ANGPTL4,* were significantly upregulated by HPGD in MB231 cells compared to controls, while these genes were downregulated by HPGD in MB436. Specifically, the expression levels of *MMP9* and *RELN* that we tested showed no significant changes by HPGD in MB436 cells, whereas those are highly increased by HPGD in MB231 cells. In contrast, genes such as *IL2RG*, *IL19RA*, and *HDAC9* were downregulated by HPGD in MB231 cells but rechanged in MB436 cells (Supplementary Figure S5). These data suggest that HPGD promotes cancer proliferation and growth in MB231 cells through modulation of the KRAS signaling pathway, a mechanism not detected in MB436 cells. This highlights a cell-specific role for HPGD in regulating tumorigenic pathways, with KRAS signaling being a key target in MB231 TNBC cells.

## 3. Discussion

Prostaglandins, particularly prostaglandin E2 (PGE_2_), have been implicated in tumorigenesis including breast cancer [4]. However, the role of HPGD, the enzyme responsible for degrading prostaglandins and maintaining homeostasis, remains controversial in breast cancer development. In this study, we investigated the functional role of HPGD in TNBC, revealing that its effects on tumor growth, proliferation, and viability are species- and cell line-dependent.

Our findings demonstrate that HPGD regulates cell growth and proliferation in human TNBC cells in a cell line-specific manner. Specifically, HPGD overexpression enhanced proliferation and growth in MB231 cells, while it decreased these parameters in MB436 cells and had no significant effect on MB468 cells. Importantly, these effects appeared to be independent of PGE_2_ levels, as supplementation with exogenous PGE_2_ did not alter cell growth or proliferation in human TNBC cells. This suggests that the observed effects of HPGD on TNBC cell growth are not mediated through its canonical enzymatic function of degrading PGE_2_. Instead, we found that HPGD expression regulated the expression of genes involved in KRAS signaling in a cell-line dependent manner. Notably, KRAS signaling was upregulated in MB231 cells, but not in MB436 cells, further supporting the hypothesis that HPGD may exert its effects on cell proliferation through non-canonical mechanisms. The regulation of KRAS signaling by HPGD represents a novel finding, which implicates HPGD’s non-enzymatic functions in modulating key oncogenic pathways in certain TNBC. HPGD blockade using small inhibitory molecules would benefit a subset of breast tumors that express high HPGD [12].

In addition to our findings in human TNBC cell lines, we also explored the functional roles of murine Hpgd in TNBC. Overexpression of Hpgd in murine TNBC cells resulted in a modest reduction in cell proliferation and growth across most of the murine cell lines tested, with the exception of 4T1.2 cells, which did not show significant changes. These results suggest that the effects of HPGD on tumor growth and proliferation are species-dependent, with a more pronounced inhibitory effect observed in murine TNBC cells compared to human TNBC cells. While human HPGD could both positively and negatively regulate cell proliferation depending on the cell line, murine Hpgd predominantly had a modest inhibitory effect on cell growth. Collectively, these findings highlight the complex and context-dependent role of HPGD in cancer biology.

In xenograft mouse models using human lung cancer cells, HPGD overexpression has been shown to decrease tumor growth, suggesting a tumor-suppressive role of the enzyme in certain contexts [17]. Interestingly, in this lung cancer model, HPGD overexpression induced epithelial-to-mesenchymal transition (EMT) in cancer cells, which contributed to tumor promotion. This phenomenon was not observed in murine TNBC cells in our study, where HPGD overexpression did not alter migratory phenotypes or induce EMT. Given that human TNBC cells exhibited diverse responses to HPGD overexpression with regard to proliferation, we chose not to investigate EMT or migration regulation in further detail. Moreover, RNA sequencing analyses did not reveal significant changes in gene expression related to EMT, invasion, migration, or metastasis, suggesting that HPGD overexpression does not significantly regulate these processes in human TNBC cells.

The role of HPGD in tumorigenesis has been reported to vary across different cancer types, further supporting the idea that its function is tissue dependent. High HPGD expression levels are associated with a good prognosis in patients with colon cancer, gastric cancer, lung cancer, and breast cancer, which has led to the hypothesis that HPGD functions as a tumor suppressor [18,19,20,21,22]. However, other studies suggest that HPGD can act as a tumor promoter, as inhibition of HPGD has been shown to increase tumorigenesis, including invasion and metastasis, through processes like EMT [23]. Collectively, these observations underscore the complexity of HPGD’s role in cancer, with its function likely depending on the tissue type, stage of the disease, and other molecular factors that modulate its activity.

Our findings using a mutant form of Hpgd, which lacks the ability to bind substrate, provide further evidence that the non-enzymatic function of Hpgd, rather than its canonical role in PGE_2_ degradation, is critical for regulating cell proliferation in TNBC. In both E0771 and EMT6 cells, the overexpression of the Hpgd mutant resulted in similar effects on cell proliferation and colony formation as the wild-type enzyme. Notably, Hpgd overexpression in EMT6 cells did not significantly alter PGE_2_ levels, reinforcing the idea that non-enzymatic functions of Hpgd, rather than its enzymatic action on PGE_2,_ are crucial for regulating cancer cell proliferation. These results suggest that the enzymatic activity of Hpgd may not be necessary for its tumorigenic effects, at least in the context of TNBC.

It is important to recognize that cellular PGE_2_ regulation is complex, involving not only HPGD but also other factors such as prostaglandin transporters located in the membrane and upstream key regulators like cyclooxygenase-2 (COX2) [24]. In our study, HPGD overexpression did not reduce intracellular PGE_2_ levels in human breast cancer cells, which may be due to compensatory mechanisms involving other regulators of PGE_2_ homeostasis. Previous research has indicated that prostaglandin receptors and transporters, including prostaglandin transporter (PGT), multiple drug resistance-associated protein 4 (MRP4), and PGE_2_ receptors (EP4), exhibit differential expression in various breast cancer cells and may play a role in maintaining PGE_2_ balance [9,23,24]. Given these complexities, further research is needed.

In summary, our findings suggest that HPGD exerts its tumorigenic effects through non-canonical mechanisms that are independent of its enzymatic function and PGE_2_ regulation. While HPGD may act as a tumor suppressor in some contexts, its role in TNBC is species- and cell line-dependent for proliferation and tumor growth. The identification of HPGD’s potential role in modulating KRAS signaling pathways further supports its involvement in cancer progression. These findings contribute to the growing body of literature on the complex and context-dependent functions of HPGD in cancer biology and highlight the need for future studies to explore its non-enzymatic roles and their implications for therapeutic strategies in TNBC.

## 4. Materials and Methods

### 4.1. Cell Lines

MDA-MB231, MDA-MB436, MDA-MB468, Py8119, 4T1.2, EMT6, and E0771 cell lines were obtained from ATCC and cultured according to the manufacturer’s instructions. The selection of cell lines includes the most representative human TNBC cells, MDA-MB231 and MDA-MB436, which are aggressive and metastatic, for this study. MDA-MB468 is another representative TNBC cell line that has BRCA1 deficiency, which is critical for TNBC phenotypes. MDA-MB231, MDA-MB436, MDA-MB468, Py8119, and E0771 cells were maintained in high glucose DMEM (Gibco, Grand Island, NY, USA) supplemented with 10% heat-inactivated FBS (Gibco, Grand Island, NY, USA) and 2% Anti-Anti (Gibco, Grand Island, NY, USA) as previously described [25]. Moreover, 4T1.2 and EMT6 cells were maintained in RPMI 1640 (Corning, Corning, NY, USA) supplemented with 10–15% heat-inactivated FBS and 2% Anti-Anti (Gibco, Grand Island, NY, USA).

### 4.2. Stable Human and Murine Cell Lines

MDA-MB231, MDA-MB436, and MDA-MB468 were treated with 5 μg/mL polybrene in media and transduced with lentiviral particles (10 MOI) carrying pLV-CMV-mGFP-T2A-puro-HPGD (Origene, Rockville, MD, USA) or controls (pLV-CMV-mGFP-T2A-puro) and followed by 10-day selection with puromycin (2 μg/mL) as previously described [25]. For murine stable cell lines, Py8119, 4T1.2, EMT6, and E0771 were treated with 5 μg/mL polybrene and transduced with lentiviral particles (100–300 MOI) carrying pLV-Hygro-CMV > mHpgd:EGFP (Vector Builder, Chicago, IL, USA) or empty vector (pLV-Hygro-CMV-EGFP) and followed by 10-day selection of hygromycin (100–500 μg/mL). To construct mutant Hpgd-expressing cell lines, E0771 and EMT6 were treated with 5 μg/mL polybrene and transduced with lentiviral particles carrying pLV-Hygro-CMV > (mutantHpgd):T2A:EGFP (Vector Builder) or control virus carrying pLV-Hygro-CMV:T2A:EGFP and followed by selection using hygromycin (100–500 μg/mL) for 7–10 days. Stable overexpression was confirmed through Western blotting.

### 4.3. Western Blot

Cells were lysed using RIPA lysis buffer (Thermo Fisher, Hend, OR, USA, 25 mM Tris HCl pH 7.6, 150 mM NaCl, 1% NP-40, 1% sodium deoxycholate, 0.1% SDS) supplemented with a protease inhibitor cocktail set III, EDTA free (EMD Millipore, Mississauga, ON, Canada) and a phosphatase inhibitor cocktail (Gold bio St. Louis, MO, USA) in ice. The protein concentration of lysed cells was determined using the BCA Protein Assay (Pierce, Rockford, IL, USA) according to the manufacturer’s instructions. A total of 25 μg of cell lysates were mixed with a 6X SDS loading buffer (Thermo Fisher) and boiled at 100 °C for 5 min to denature the proteins before loading. Whole cell lysates were separated on a 10% SDS gel and transferred to 0.45 µm nitrocellulose membranes (Invitrogen, Carlsbad, CA, USA) for further blotting. Membranes were blocked with blocking buffers (3% BSA solution or 5% skim milk) for 1 h at room temperature and incubated overnight at 4 °C with primary antibodies in blocking buffers: anti-15-PGDH antibody [EPR 14332-19] (Abcam, Waltham, MA, USA, 1:1000), anti-GFP antibody (Sigma Aldrich, Saint Louis, MO, USA, 1:5000), and anti-beta-actin antibody (Sigma-Aldrich, Saint Louis, MO, USA, 1:5000). Membranes were washed with TBST buffer (Pierce Biotechnology, Waltham, MA, USA: 25 mM Tris, 0.15 M NaCl, with 0.05% Tween-20, pH 7.5) for thirty minutes, at ten-minute intervals. Membranes were then incubated at room temperature for one hour with secondary anti-rabbit IgG, HRP-linked antibody (Cell Signaling, Danvers, MA, USA, 1:1000), and anti-mouse IgG, HRP-linked antibody (Cell Signaling 1:1000). After washing, membranes were developed using ECL Western blot substrate (Pierce Biotechnology, Waltham, MA, USA) on the iBright Imager (Thermo Fisher, Hend, OR, USA).

### 4.4. Colony Formation

TNBC cells (5 × 10^4^ per well for human cells and 1 × 10^4^ per well for murine cells) were plated in a 6-well plate and incubated for 7–10 days. For visualization, colonies were stained using crystal violet (0.2%) for 5 min and washed twice with tap water. Colony formation units (CFUs) were quantified using the Image J version 1.54 imaging software (Bethesda, MD, USA) by adjusting threshold to highlight colonies, watershed function to outline colonies and analyze particles to count colonies restricted to those >100 pixels.

### 4.5. Cell Viability Assays

The MTT (3-(4,5-dimethyulthialzol-2-yl)-2,5-diphenyltetrazolium bromide) assays were performed to measure cellular viability. Cells (5 × 10^3^ cells per well) were plated in 96-well plates for 48 h. After removing culture media from cells, MTT solution (5 mg/mL, EMD Millipore, Mississauga, ON, Canada)) was added and incubated for 2 h. Following removal of the MTT solution, DMSO (100 μL) was added to each well and plate absorbance was read at 500 nm using the Victor3 plate reader (Thermo Fisher, Hend, OR, USA). The Calcein AM assays were performed by treating cells in 96-well plates with Calcein AM in PBS (1 μM, Invitrogen, Carlsbad, CA, USA) and incubated for 1 h at 37 °C, after which fluorescence was recorded using the Victor3 plate reader.

### 4.6. Three-Dimensional Spheroid Culture

Human TNBC cells (5 × 10^3^ cells per well) were suspended in MammoCult™ Basal Medium (Stemcell Technologies #05620, Vancouver, Canada), supplemented with 10% MammoCult™ Proliferation Supplement (Stemcell Technologies, Vancouver, BC, Canada), and placed in low-attachment 6-well plates. Spheroid culture was left undisturbed for 10 days and spheroids were imaged at 10× magnification of the EVOS M5000 Imaging System (Invitrogen, Carlsbad, CA, USA). Spheroid number and diameter were quantified using 3–4 independent fields of view per well. The total number of cells in the spheroid was quantified by spinning spheroids down and resuspending them in PBS into single cells that were quantified using the Countess 3 Automated cell counter (Invitrogen, Carlsbad, CA, USA).

### 4.7. Live Imaging of Cell Growth

Cells (5–8 × 10^3^ cells per well) were seeded in a 96-well plate and monitored for cell confluency every 4 h using the Incucyte live cell imager (Sartorius, Gottingen, Germany). Incucyte software (v2020C Sartorius, Gottingen, Germany) was used to calculate average confluency per well and per condition with ± SEM reported for the average.

### 4.8. PGE_2_ ELISA

PGE_2_ ELISA was performed using a Prostaglandin E_2_ ELISA Kit—Monoclonal protocol (Cayman #514010, Ann Arbor, MI, USA). For intracellular PGE_2_ quantification, cells seeded for 24 h to 80% confluency in a 100 mm dish were lysed through sonication (20% amps, 5–6 times of sonication at 4 °C for 5 s) and whole cell lysates were resuspended in 100–200 μL of PBS. For human cell lines, the lysate sample was diluted 1:2 and 1:10. For murine cell lines, lysate was diluted 1:25 and 1:50. Whole cell lysates were then used for quantification along with PGE_2_ standards according to the manufacturer’s protocol. Lysate protein concentration was measured using BCA Protein Assay (Pierce, Rockford, IL, USA), and PGE_2_ levels were standardized to protein levels.

### 4.9. qRT-PCR

Total RNA was isolated from the cells using Trizol (Invitrogen) according to the manufacturer’s protocol and used for reverse transcriptase (RT) reaction with a High Capacity cDNA Reverse Transcription Kit (ThermoFisher 4368814, Thermo Fisher, Hend, OR, USA) to construct the cDNA library. Real-time qPCR was performed using PowerUp SYBR Green master mix (Applied Biosystems A25741, Foster City, CA, USA) and the Cfx384 Real-time PCR (Bio-Rad, Hercules, CA, USA). Cq values were normalized relative to the expression of the endogenous control gene 36B4 using the 2^(−ΔΔCq)^ method and plotted. The primer pairs (5′–3′) used are;

RELN-F: GCCACTTCTTGTGGTGACCT,
RELN-R: TGCCAGGAATCCGATCTTGC,
PTGS2-F: CAAATTGCTGGCAGGGTTGC,
PTGS2-R-AGGGCTTCAGCATAAAGCGT,
BMP2-F: CTGCGGTCTCCTAAAGGTCG,
BMP2-R: CTGCGGTCTCCTAAAGGTCG,
IL1B-F: CAGAAGTACCTGAGCTCGCC,
IL1B-R: GGTCCTGGAAGGAGCACTTCA,
IGF2-F: CGCTGTTCGGTTTGCGAC,
IGF2-R: TTCCCATTGGTGTCTGGAAGC,
MMP9-F: TTCAGGGAGACGCCCATTTC,
MMP9-R: AACCGAGTTGGAACCACGAC,
ANGPTL4-F: CCACTTGGGACCAGGATCAC,
ANGPTL4-R: AAACCACCAGCCTCCAGAGA,
CCND2-F: GAACCTGGCAGCTGTCACTC,
CCND2-R: TGGCAAACTTAAAGTCGGTGGC,
IL2RG-F: TTTCTGGCTGGAACGGACG,
IL2RG-R: GCCAGTCCCTTAGACACACC,
CXCR4-F: AGGTAGCAAAGTGACGCCG,
CXCR4-R: GCGGGGCATTTTCACTGATCC,
SPP1-F: ACAAATACCCAGATGCTGTGGC,
SPP1-R: ACTTGGAAGGGTCTGTGGGG,
IL10RA-F: CAGACGCTCATGGGACAGAG,
IL10RA-R: GGTGTCCAGTGGAGGATGTG,
IL7R-F: TCCAACCGGCAGCAATGTAT,
IL7R-R: AGGATCCATCTCCCCTGAGC,
TNFAIP3-F: AGAGAGATCACACCCCCAG,
TNFAIP3-R: TGCTCTCCAACACCTCTCC,
HDAC9-F: GCTGTTTGGAGAAGGGGGAG,
HDAC9-R: TGGCAGAGGAGAAACCTCCG,
GFPT2-F: CTCGAGCCATCCAGACCTTG,
GFPT2-R: CACCCAGGAGCACTGTGTTG,
ETS1-F: AAAGAGACCACAGACTTTGAGGG,
ETS1-R: TCTGCTCTCAGCACCTCACTTA.

### 4.10. Animal Experiment

Animals were maintained in an AAALAC-accredited facility in accordance with the Guide for the Care and Use of Laboratory Animals. All procedures for animal use were approved by the GWU Institutional Animal Care and Use Committee. Athymic nude mice (6–8 weeks old females, Charles River) were orthotopically injected with human breast cancer cells (MDA-MB231-Control-EGFP, MDA-MB231-HPGD-EGFP, 2 × 10^6^ cells per mouse) in the 4th mammary fat pads. Tumor volumes were measured weekly using calipers and the body weight of mice were monitored weekly. Tumor volume was calculated using an equation = length × (diameter)^2^ × 0.4. 

### 4.11. Statistical Analysis

Viability assays, colony formation, growth assays, three-dimensional spheroid assays, and PGE_2_ assays were analyzed to compare the values measured in control groups to those of overexpressed HPGD. Statistical significance performed using a two-tailed Student’s *t*-test analysis on GraphPad Prism 10 software. *p*-values indicate that * *p* < 0.05, ** *p* < 0.01, *** *p* < 0.001, and **** *p* < 0.0001.

## Data Availability

Sequencing data are available to the public on NCBI. The datasets used and/or analyzed during the current study are available from the corresponding authors upon reasonable request.

## Supplementary Materials

The supporting information can be downloaded at: https://www.mdpi.com/article/10.3390/ijms26051912/s1.
