# Supplementary material for: Tumor Regulatory Effect of 15-Hydroxyprostaglandin Dehydrogenase (HPGD) in Triple-Negative Breast Cancer
Source: Int J Mol Sci. 2025 Feb 23;26(5):1912. doi: 10.3390/ijms26051912 (PMC11899648; doi:10.3390/ijms26051912)

Supplementary Figure S1. HPGD regulates proliferation of human TNBC cells in a cell line-dependent manner. (A) Representative western blot of ectopic HPGD expression using lentiviral transduction followed by 10 days of puromycin selection (2μg/ml) in human TNBC cell lines. (B) Growth (% confluency) of the HPGD-overexpressing (HPGD-OE) and control TNBC cells.

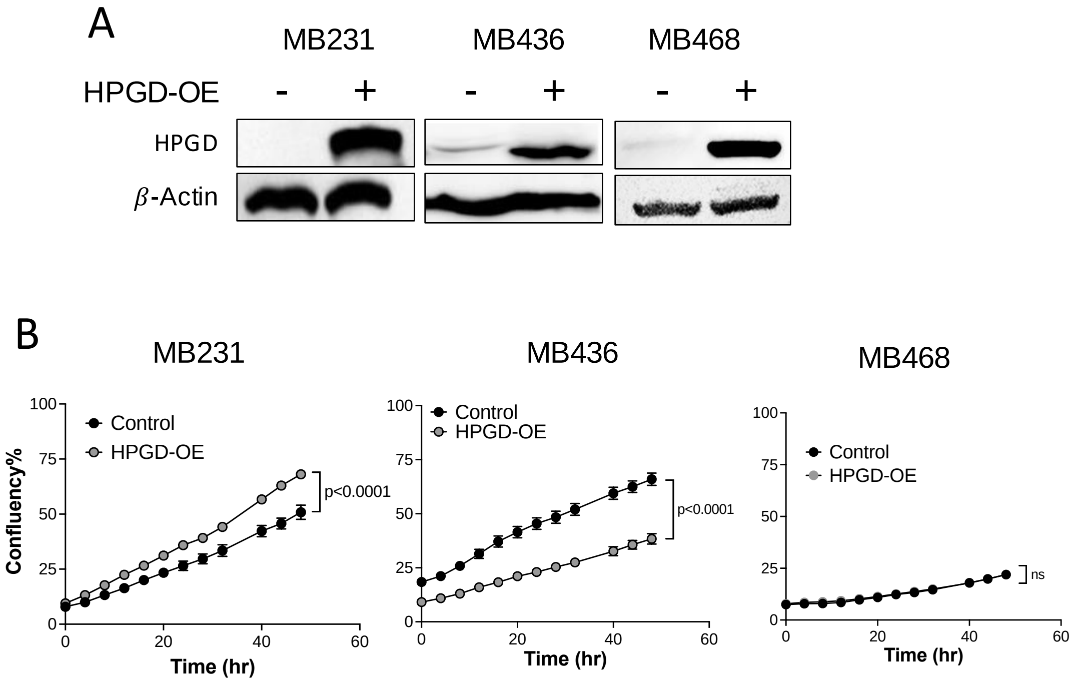

Supplementary Figure S2. Hpgd slightly decreases viability of murine TNBC cells. (A) Viability (%) of Hpgd-OE cells for 48 hours by MTT assay (n=6 biological replicates). (B) Growth (% confluency) of Hpgd-OE and control cells (n=6 biological replicates). (C) Colony formation of Hpgd-OE and control cells (n=3-6 biological replicates) for 10-12 days after seeding. (D) Wound density (%) by Py8119-Hpgd and 4T1.2-Hpgd cells using scratch wound assays up to 48 hours (n=6 biological replicates). Data are mean±SD. p-values using a two-tailed paired Student's t-test.

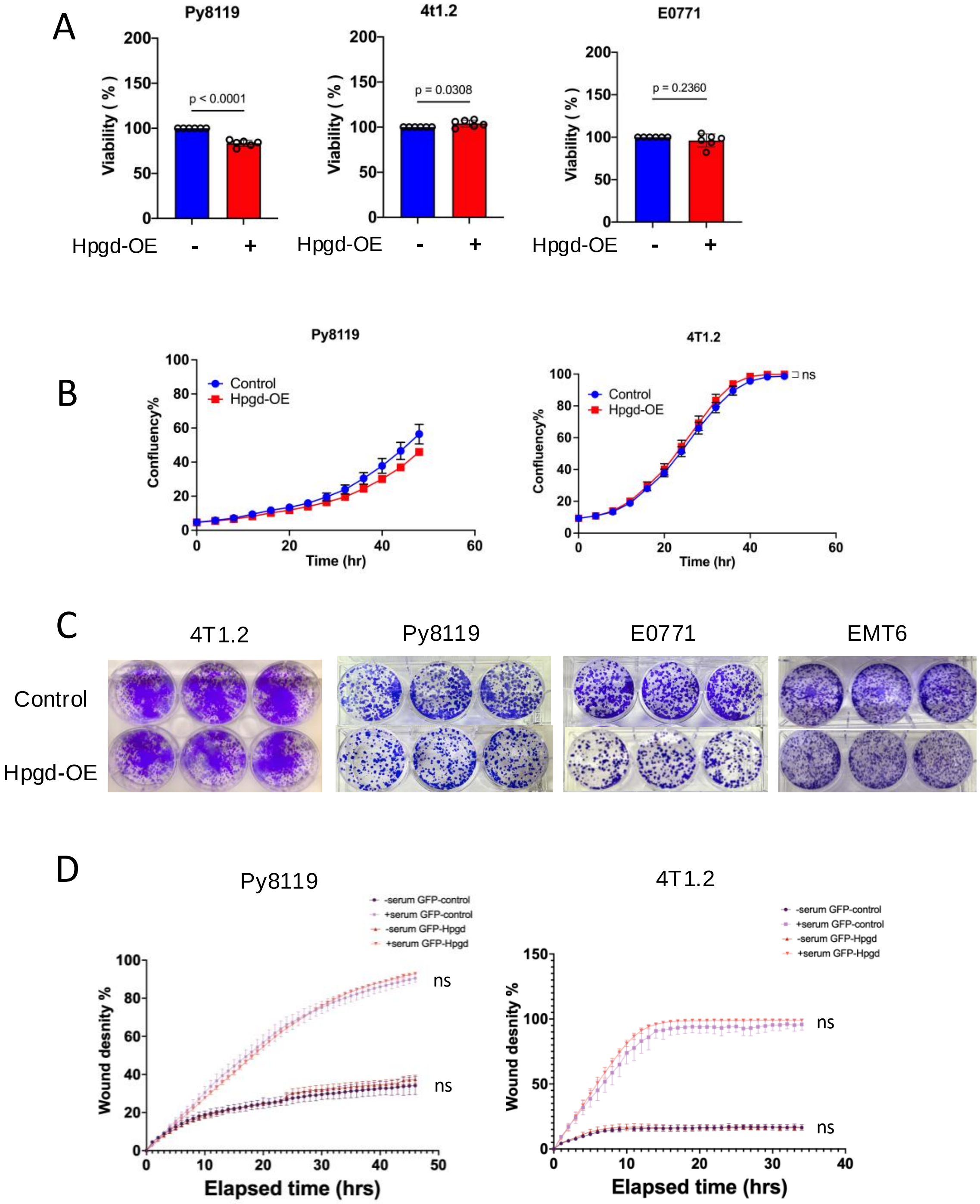

Supplementary Figure S3. Human HPGD regulates spheroid formation of TNBC cells in a cell line-dependent manner and murine Hpgd does not regulate mammosphere formation of murine TNBC cells. (A) Representative microscopic images of spheroids formed for human TNBC HPGD-OE and control cells in 3D mammosphere media for 10 days and imaged under a light microscope. (B) Total number of spheroids, average diameters of spheres, and average number of cells from total spheroids of Py8119-Hpgd or 4T1.2-Hpgd and their control cells. (n=3-6 biological replicates). (C) Intracellular PGE_2 levels of HPGD-expressing and control TNBC cells. PGE_2 levels normalized to whole cell protein concentration (n=3 technical replicates). Data are mean±SD, p-values using a two-tailed Student's t-test.

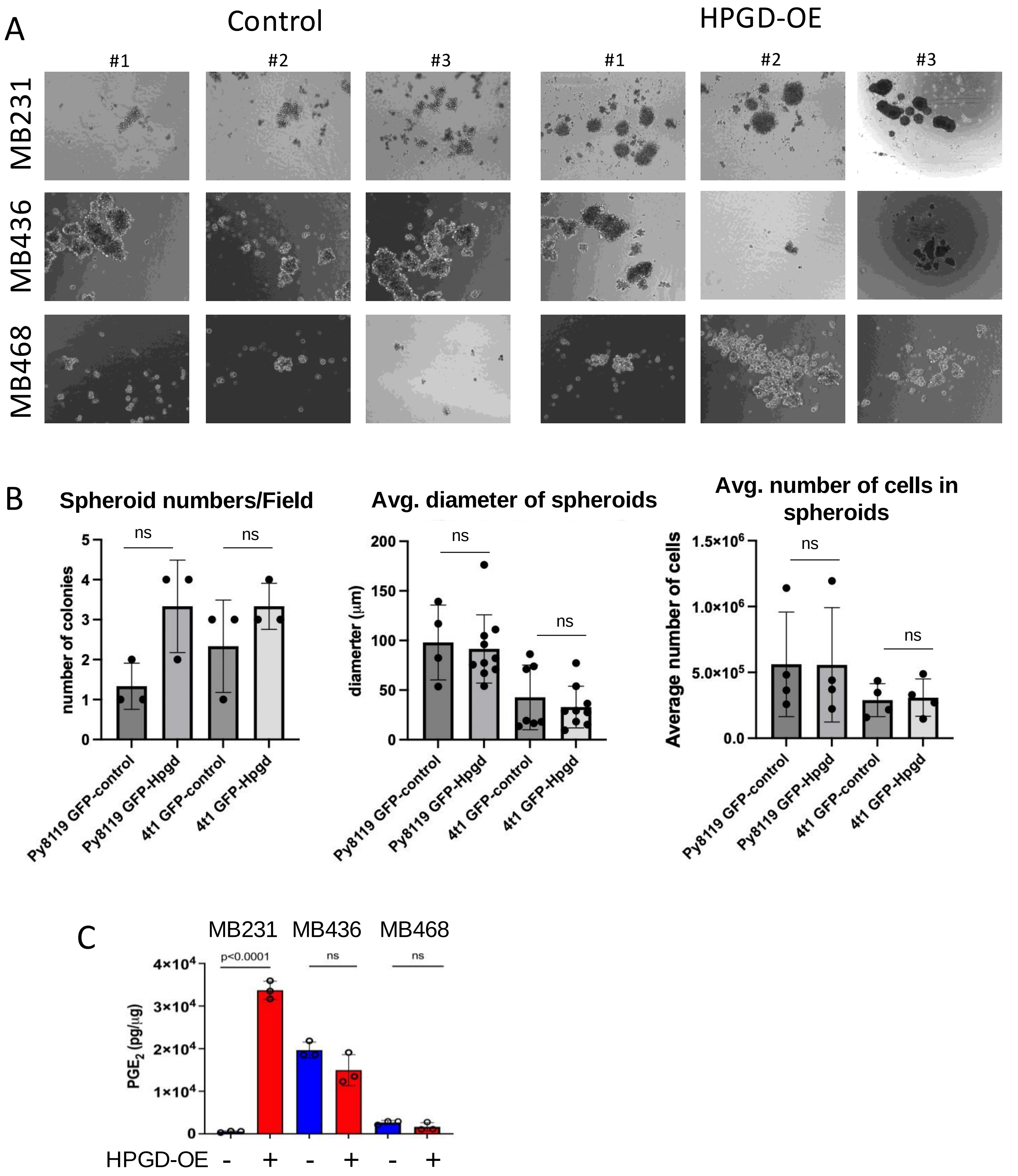

Supplementary Figure S4. The enzymatic function of Hpgd is not required for regulation of cell proliferation. (A, B) Viability (%) and growth (Confluency %) of cells-overexpressing Hpgd^{WT}, Hpgd^{MUT}, or control vector using MTT assays and Incucyte imaging system (n= 6 biological replicates). (C) Colony forming assays of Hpgd^{WT}, mutant Hpgd^{MUT}, and control vector-expressing cells and parental cells in E0771 and EMT6. Data are mean$\pm$SD. p-values using a two-tailed Student's t-test.

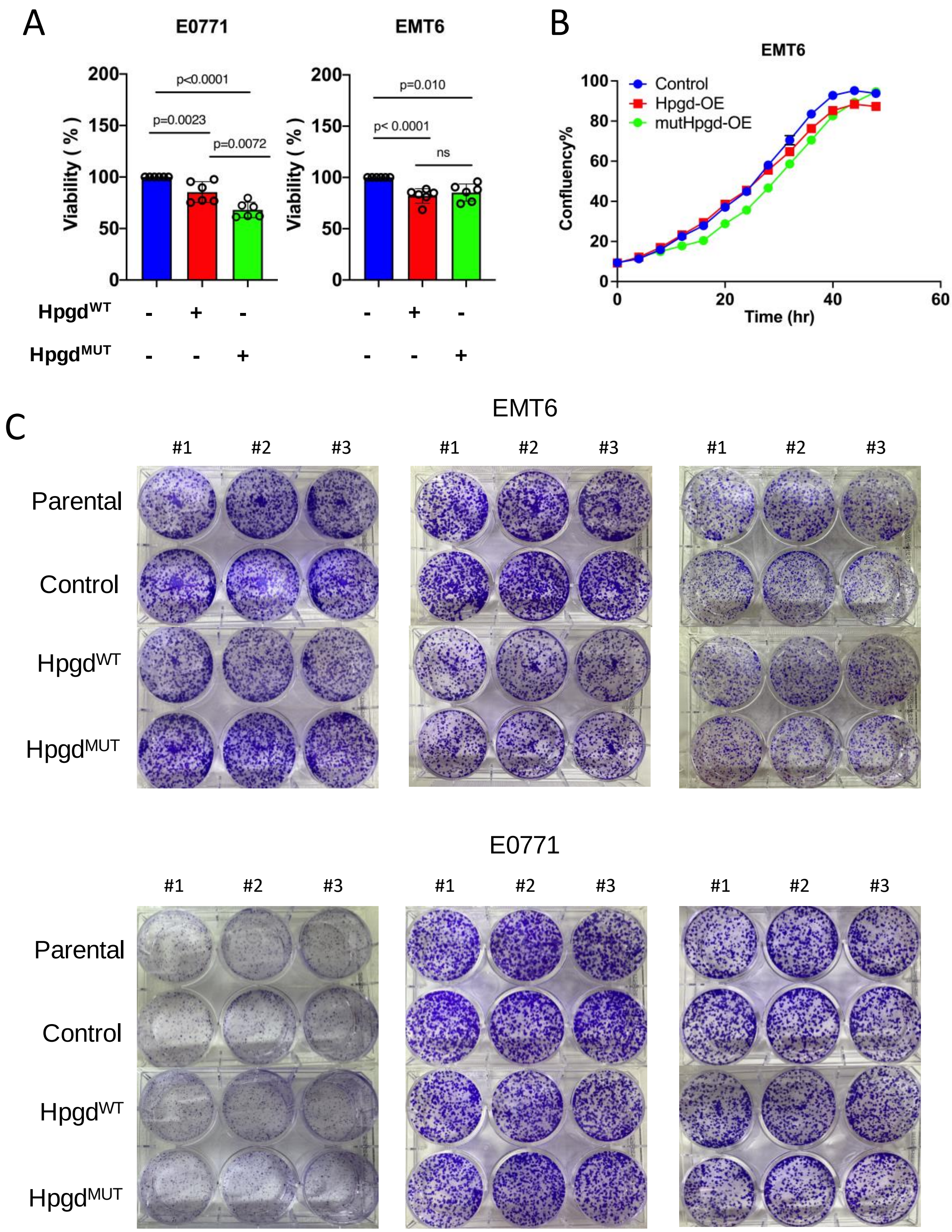

Supplementary Figure S5. HPGD regulates Kras signaling differently in MB231 or MB436 cells. (A) Relative mRNA levels of genes involved in Kras signaling pathways y qRT-PCR. P-values by unpaired t-test.

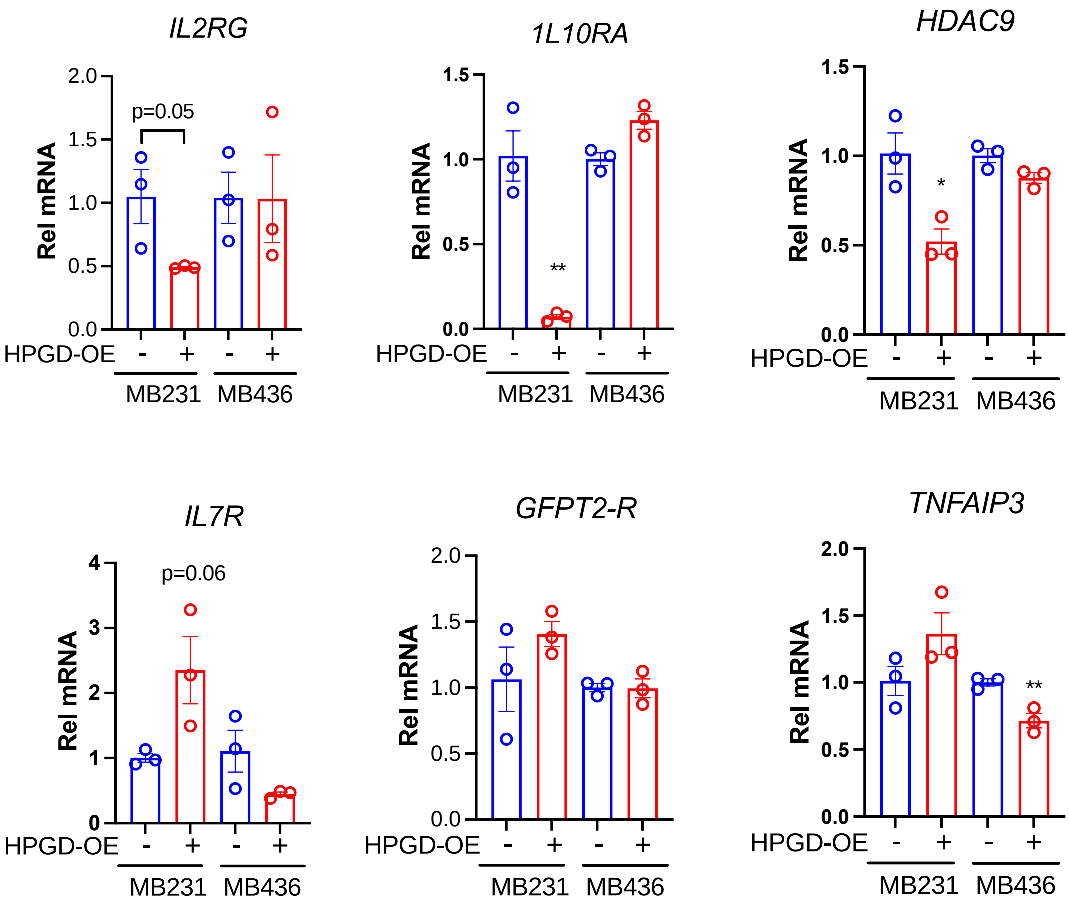

Supplement: Supplementary file 1 [file ijms-26-01912-s001.zip › ijms-3455212-supplementary.pdf]
